# Predicting infarction growth rate II using ANFIS-based binary particle swarm optimization technique in ischemic stroke

**DOI:** 10.1016/j.mex.2023.102375

**Published:** 2023-09-13

**Authors:** Afnan Al-Ali, Uvais Qidwai, Saadat Kamran

**Affiliations:** a Computer Science and Engineering Department, Qatar University, Doha, Qatar; b Department of Neurology, Hamad Medical Corporation, Doha, Qatar

**Keywords:** ANFIS-BPSO based Prediction System, Ischemic stroke, Adaptive neuro-fuzzy inference system (ANFIS), Binary particle swarm optimization technique (BPSO), Infarction growth rate II (IGR II)

## Abstract

Ischemic stroke, a severe medical condition triggered by a blockage of blood flow to the brain, leads to cell death and serious health complications. One key challenge in this field is accurately predicting infarction growth - the progressive expansion of damaged brain tissue post-stroke. Recent advancements in artificial intelligence (AI) have improved this prediction, offering crucial insights into the progression dynamics of ischemic stroke. One such promising technique, the Adaptive Neuro-Fuzzy Inference System (ANFIS), has shown potential, but it faces the 'curse of dimensionality' and long training times as the number of features increased. This paper introduces an innovative, automatic method that combines Binary Particle Swarm Optimization (BPSO) with ANFIS architecture, achieves reduction in dimensionality by reducing the number of rules and training time. By analyzing the Pearson correlation coefficients and *P*-values, we selected clinically relevant features strongly correlated with the Infarction Growth Rate (IGR II), extracted after one CT scan. We compared our model's performance with conventional ANFIS and other machine learning techniques, including Support Vector Regressor (SVR), shallow Neural Networks, and Linear Regression.

• Inputs: Real data about ischemic stroke represented by clinically relevant features.
• Output: An innovative model for more accurate and efficient prediction of the second infarction growth after the first CT scan.
• Results: The model achieved commendable statistical metrics, which include a Root Mean Square Error of 0.091, a Mean Squared Error of 0.0086, a Mean Absolute Error of 0.064, and a Cosine distance of 0.074.

## Graphical abstract

**Figure:**
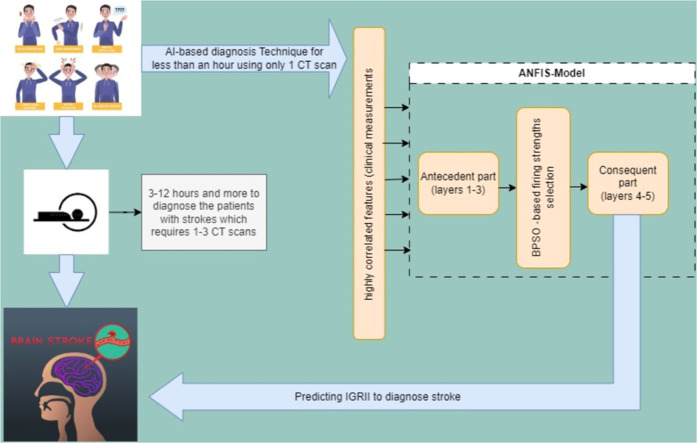

Specifications table

**Table:** 

Subject area:	Engineering
More specific subject area:	Applied AI for Health Informatics
Name of your method:	ANFIS-BPSO based Prediction System
Name and reference of original method:	N. A
Resource availability:	N. A

**Method details**

## Background

A stroke occurs when a blood artery in the brain ruptures or is blocked, leading to a lack of blood flow to a specific brain area. As a result, parts of the brain may suffer damage or degeneration. Stroke victims may experience permanent brain damage, chronic disability, or even death [1]. The occurrence of stroke can be classified into two main types: ischemic and hemorrhagic. Ischemic stroke, the more prevalent type, is characterized by a blockage of a major blood vessel in the brain caused by either a blood clot or the accumulation of fatty deposits and cholesterol, also known as plaque. On the other hand, hemorrhagic stroke results from a rupture of a blood vessel in the brain, leading to the leakage of blood into surrounding tissues. In this case, pressure builds up in the nearby brain tissue, causing additional damage and irritation [2].

The risk factors for stroke can be categorized into those that can be changed, treated, or medically managed and those that cannot. The modifiable risk factors for stroke include high blood pressure, heart disease, diabetes, smoking, birth control pills, high red blood cell count, high blood cholesterol and lipids, lack of exercise, obesity, excessive alcohol use, illegal drugs, abnormal heart rhythm, and cardiac structural abnormalities. Among these, high blood pressure is considered the most significant risk factor, as it can damage the arteries supplying blood to the brain. Meanwhile, the non-modifiable risk factors for stroke include older age, race, gender, history of prior stroke, and heredity or genetics [[3], [4]].

A stroke diagnosis typically involves physical examinations and imaging studies of the brain. Doctors perform various tests to gather information about the symptoms of the patient suspected to have a stroke. Even if the symptoms of a stroke are clear, brain imaging is necessary to determine the cause of the stroke, which area of the brain has been affected, and the severity of the stroke [5]. To ascertain the positive impact of putative treatments on the outcome of strokes, measuring the extent of infarcted brain tissue is thus a crucial component of preclinical research. The existence of edema throughout the acute and subacute phases of ischemia damage is a well-known issue when estimating the real infarction volume in some animal studies. A direct assessment of the infarction volume is only somewhat reliable since edema induces swelling of the brain tissue, which overestimates the real amount of the infarction because edema must be considered when calculating infarction volume during the acute phase. Concerning these studies, during a significant period of time, the pace of the volumetric growth rate remains relatively constant, and it is possible to predict any increase in volume with a reasonable level of precision within a predetermined time frame [6].

A CT (computerized tomography) scan is considered the most widely utilized imaging method to measure this rate. However, it is unknown when imaging would be most effective in determining the infarction's size. Very early imaging carries a risk since it can underestimate the amount of the stroke and uses multiple X-ray images to create a 3-dimensional image of the brain to help doctors identify any areas of concern [[5], [7]].

In the current literature, a research gap exists in providing a reliable method to estimate the infarction volume during the acute phase of a stroke, as the presence of edema can lead to inaccurate estimations using traditional imaging techniques. Additionally, the optimal timing for imaging to determine infarction size remains unclear. This study aims to address these gaps by developing a model that can accurately predict the infarction growth rate (IGR) at a specific time, thereby reducing the need for multiple CT scans and better understanding the stroke's severity and affected brain areas. Besides, the lack of medical datasets would strict the use of the current AI techniques based mainly on deep learning as they require large amounts of data for their training; Rules-based techniques such as Adaptive Neuro Fuzzy Inference System (ANFIS) sound like successful alternative methods. For this reason, this paper proposes a new rule-based model by integrating Binary Particle Swarm Optimization technique (BPSO) with ANFIS to predict the second Infarction Growth Rate (IGR II) which can be determined after the first round of CT scan, this will speed up the process of diagnosing and saves cost. This can be done by selecting 5 highly correlated features with the IGR II based on calculating the *P*-value and the Pearson correlation coefficient between each feature and the IGRII. These features represent clinical measurements comprise patient information gathered from a pooled Decompressive Hemicraniectomy database described in detail in the next section of dataset.

### Dataset

The dataset utilized in this study is similar to the one used in [[7], [8]], which is approved based on the Neurologist's opinion. It consists of 204 records with 11 characteristics. This dataset comprises patient information gathered from a pooled Decompressive Hemicraniectomy database, the components of which were received from three referral centers in three distinct countries, namely Qatar, the United Arab Emirates, and Pakistan.

Only patients with three brain CT scans and signs of acute ischemia were considered. These specifics include the patient's age, whether they have diabetes, whether they did Hemicraniectomy, their hypertension status, whether they have Dyslipidemia, blood pressure readings, INFARCT VOLUME 1 and 2, and the First infarction growth rate per hour. All these features are described in Table 1 in detail with their meanings, range of values, the *P*-value, and Pearson correlation coefficient with our target, the second infarction growth rate (IGRII).

**Table 1:** Description of the dataset.^1^

Item	Feature	Description	Values	*P*-value	Correlation with IGR2
1	AGE	AGE OF THE PATIENT	in years	0.8058	-0.0187
2	SBP	Systolic blood pressure reading	-	0.9253	0.0071
3	DBP	Diastolic blood pressure reading	-	0.6222	0.0374
4	HTN	Hypertension diagnosis	0 – Absent,1 - Present	0.9350	0.0062
5	DM	DIABETES MELLITUS	0 – Absent, 1 - Present	0.3811	0.0664
6	**DYSLIP**	DYSLIPIDEMIA	0 – Absent, 1 -Present	0.1750	-0.1027
7	**UNCAL**	UNCAL HERNIATION PRESENT	0 – Absent, 1 -Present	0.0312	0.1625
8	**TEMPORAL**	TEMPORAL LOBE INVOLVED	0 – Absent, 1 -Present	0.0105	0.1926
9	**INFVOL1**	INFARCT VOLUME 1 (cm3)	-	0.0100	-0.1936
10	INFVOL2	INFARCT VOLUME 2 (cm3)	-	6.3804e-09	0.4202
11	**Growthrate_1**	First infarction growth rate per hour	-	2.1920e-33	0.7525

1 The bolded features represent the ones with the least *P*-values and highest correlation coefficients with the IGR II

### Methodology

The first step in our methodology is removing the null and missing values from our dataset. To do so, we used MATLAB function rmmissing, which identifies any missing values in the data and removes the entire row if it contains at least one missing value [9]. After this cleaning step, the number of records we have collected is 177 patients. As shown in Table 1, we calculated the *P*-value and the correlation coefficient between each feature and our target. This calculation helped us to choose the most significant features that impact predicting the IGR II. The final set of features selected for our model is (DYSLIP, UNCAL, TEMPORAL, INVOL1, and Growthrate_1) and they have been shown in bold in Table 1. For all these features as noticed from the table the least *P*-value is close to its threshold of 0.05 and the highest correlation coefficient of absolute 0.1. Regarding the 'DYSLIPIDEMIA' feature, based on a study in 2022 [10] this feature is a major risk factor for coronary heart disease but its impact on ischemic stroke is still under discover, so having *P*-value very close to the threshold of *P*-values motivated us to add this feature to the set of selected features. For those features which need normalization, they were normalized in the range of 0,1 to unify the range of their values. We excluded INVOL2 (Infarct Volume 2) because this feature can be extracted after the second CT scan round, which is not considered for this study.

**Fig. 1:**
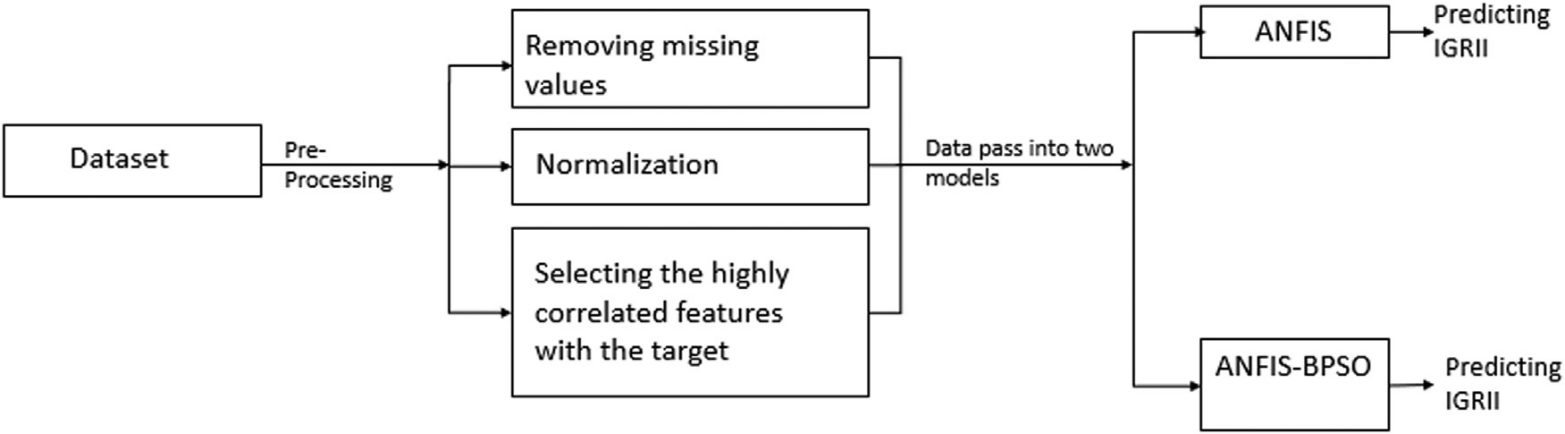
The experiments block diagram using both models.

**Fig. 2:**
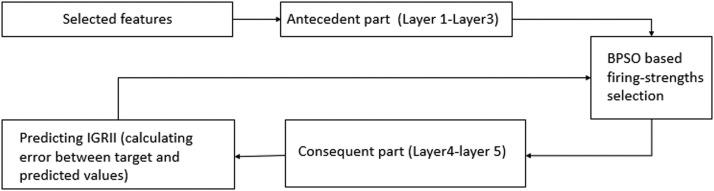
The block diagram of ANFIS-BPSO.

After preparing the dataset in the form ready to be input to our models, two types of rule-based machine learning techniques have been tested: the conventional ANFIS [11] and a modified version of it by embedding the BPSO as a feature selector in its architecture. This modification of the second model aims to reduce the number of generated rules and the training time and improve performance. Fig. 1 shows the block diagram of our methodology, and Fig. 2 shows the block diagram of ANFIS-BPSO.

## Adaptive neuro fuzzy inference system (ANFIS)

ANFIS is a hybrid neural network that combines fuzzy logic and neural networks. It comprises two main parts, the antecedent and the consequent, forming the fuzzy rules that make up the network. During training, the parameters of each section are updated using a hybrid optimization technique. The ANFIS structure consists of five layers, with the antecedent part spanning the first three layers and the consequent part spanning the remaining layers. The first layer is the fuzzification layer which calculates membership degrees for each input and updates the antecedent parameters using the gradient descent algorithm. The second one is the rule layer which calculates the firing strength of each rule, and the normalization layer is the third layer which normalizes the firing strengths using min-max normalization. The fourth layer is the defuzzification layer which marks the beginning of the consequent part and updates its parameters using the least square error technique in the forward path. The output layer, the fifth layer, sums up the outputs of the fourth layer. ANFIS updates its parameters using a two-pass hybrid learning algorithm that combines backward and forward updates [11].

The Adaptive Neuro-Fuzzy Inference System (ANFIS) has a broad range of applications within the medical domain, particularly in the diagnosis of various diseases, including diabetes [12] and cancer [[13], [14]]. Importantly, its efficacy has been demonstrated in stroke diagnosis, as evident in studies such as [[15], [16]]. This paper further explores the utility of the ANFIS model, specifically aiming to predict the IGR II after a single round of CT scanning. After the pre-processing stage which includes: (removing missing values, normalization, and selecting the highly correlated features after calculating the *P*-value and the Pearson correlation coefficients between each one and the target), these features will pass to the ANFIS model as shown in Fig. 1 and pass to the second model ANFIS-BPSO in another experiment as will be explained later.

### ANFIS-BPSO

ANFIS uses three common fuzzy rule generation techniques: grid partitioning, subtractive clustering, and fuzzy c-mean. This paper focuses on grid partitioning, which involves splitting the data space into rectangular subspaces based on the number of membership functions, producing the best results in terms of accuracy but increasing computational time due to many tunable parameters. The grid partitioning method automatically generates all possible rules, including relevant and irrelevant rules [[17], [18]]. However, our research found that generating a complete set of fuzzy rules using grid partitioning yields the best performance when used with ANFIS compared to other data-splitting methods. Given the objective of achieving optimal performance, generating the maximum possible rule set was pursued. Concurrently, efforts were made to reduce the dimensionality, a common challenge in such scenarios. To mitigate this issue, we applied this model that harmoniously combines (ANFIS) with the (BPSO). BPSO, functioning akin to feature selection, has been employed in various studies as a robust tool. Prominent examples of its application in literature include [[19], [20], [21]]. In our model, we analyzed the ANFIS architecture, and based on the analysis, we found that the firing strengths indicate the true impact of the inputs on the outputs, compensating for any internal features. Reducing these firing strengths means decreasing the number of generated rules. To achieve this purpose, we inserted the BPSO features selection technique between the antecedent and consequent parts, as shown in Fig. 2. In each iteration, a single set of candidates’ firing strengths is tested for all samples, and the error is calculated. The candidate set of firing strengths that results in the minimum error is then used to evaluate the test set.

The following steps outline the basic approach to using BPSO as a feature selector [22]:

• Swarm Initialization: Generate a swarm of particles, each representing a unique subset of features as potential solutions to the problem.
• Fitness Function: Define a fitness function, such as model accuracy, that evaluates the quality of the feature subsets. Our study used the mean squared error between the predicted value using the ANFIS classifier at layers 4 and 5 and the original target.
• Update Particles: Move particles in the search space based on their personal best position (Pbest) and the global best position (Gbest). This movement in BPSO is driven by a transfer function converting the PSO's continuous output into binary values.
• Velocity Update: Adjust the velocity, incorporating both the particle's (Pbest) and the swarm's (Gbest), to guide particles toward potentially better search spaces.

The equation for the velocity update in Eq. (1)

(1) $$v_{i}\left(t+1\right)=w*v_{i}\left(t\right)+c_{1}*r_{1}*\left(\text{Pbest}-x_{i}\left(t\right)\right)+c_{2}*r_{2}*\left(\text{Gbest}-x_{i}\left(t\right)\right)$$

where:

– $v_{i}\left(t+1\right)$ is the velocity of the $i^{\text{th}}$ particle at ${\left(t+1\right)}^{\text{th}}$ iteration.
– w is the inertia weight.
– $v_{i}\left(t\right)$ is the velocity of the$\;i^{\text{th}}$particle at $t^{\text{th}}$ iteration.
– $c_{1}$ and $c_{2}$ are cognitive and social learning factors, respectively.
– $r_{1}$ and $r_{2}$ are two random numbers between 0 and 1.
– Pbest and Gbest are the personal best and global best positions at $t^{\text{th}}$ iteration, respectively.
– $x_{i}\left(t\right)$ is the position of the $i^{\text{th}}$ particle at $t^{\text{th}}$ iteration.
  • Position Update: Use a sigmoid function to convert velocities into probabilities, then generate a random binary number for each particle's dimension. If the random number is less than the sigmoid of the velocity, that dimension is set to 1; otherwise, it's set to 0. The equation for the position update in Eqs. (2) and (3)
    (2) $$\text{Sigmoid}\left(v_{i,d}\left(t+1\right)\right)=\frac{1}{1+e^{-v_{i,d}\left(t+1\right)}}$$
    (3) $$x_{i,d}\left(t+1\right)=\left\{ \begin{array}{cc} 1, & if\;r3<Sigmoid\left(v_{i,d}\left(t+1\right)\right)\\ 0, & otherwise\; \end{array}\right.$$

*d* is the swarm dimension, r3 is a random number between 0 and 1

• Iteration: Repeat the update steps until a stopping criterion is met, such as a maximum number of iterations, minimum error threshold, or lack of significant improvement in (Gbest).
• Best Subset Selection: Upon algorithm termination, the particle at the (Gbest) position is chosen as the best feature subset.

### Model validation and analysis

Our dataset has been split into 80 % training and 20 % testing, and by using 5-fold cross-validation, two experiments have been adopted. The first is predicting the IGR II using the conventional ANFIS and the second is predicting the IGR II using ANFIS-BPSO, both based on highly correlated features. Our experiments achieved two main points: Firstly is to predict Infarction Growth Rate (IGR) after the first round of CT scans by utilizing some clinical measurements that exhibit a high correlation with the target variable. Secondly, is the improvement in performance that ANFIS-BPSO achieved over the conventional ANFIS when using the same dataset, same parameter values, and same conditions (which are represented by the membership function being 2, the type of membership function being the Generalized-Bell shape, and the number of epochs being equal to 150). This achievement was in terms of several evaluation metrics. They are the Mean Square Error (MSE), a popular technique used to evaluate model performance by calculating the average of the squares of the difference between each model output and its desired output. The Root Mean Square Error (RMSE) authorizes large number deviations and punishes large errors, providing higher weight than MSE. This measure is crucial for predicting health-related outcomes, where utmost accuracy is necessary while avoiding even minor errors. We also considered the Mean Absolute Error (MAE) as an evaluation metric. MAE calculates the average absolute difference between each model output and its desired output. Finally, the Cosine distance evaluation metric is also included. This calculates the pairwise separation between two observations or vectors, representing this work's predicted and actual output. The comparison vectors are more similar the closer the value is to 0. The mathematical representation of each evaluation method is represented in the equations below [23].

(4) $$\text{MeanSquaredError}\left(\text{MSE}\right)=\frac{1}{n}\sum_{i=1}^{n}{\left({\hat{y}}_{i}-y_{i}\right)}^{2}\;$$

(5) $$\text{RootMeanSquareError}\left(\text{RMSE}\right)=\sqrt{\frac{1}{n}\sum_{i=1}^{n}{\left({\hat{y}}_{i}-y_{i}\right)}^{2}\;\;}$$

(6) $$\text{MeanAbsoluteError}\left(\text{MAE}\right)=\frac{1}{n}\sum_{i=1}^{n}\left|{\hat{y}}_{i}-y_{i}\right|$$

(7) CosDistance=1−(CosineSimilarity)

(8) $$\text{CosSimilarity}={\frac{{\hat{y}}_{i}y_{i}}{{∥{\hat{y}}_{i}∥}_{2}∥y_{i}∥}}_{2}$$

where *n* is the number of samples, $y_{i}\;$is the actual value of the target variable for the $i^{\text{th}}\;$sample, and ${\hat{y}}_{i}$ is the predicted value of the target variable for the *i*-th sample. In addition to the above-mentioned metrics, we considered for our comparison the number of generated rules in both experiments, the training time, the *p*-value, and the correlation between the predicted value and the actual IGR II. Table 2 shows the results of both models.

**Table 2:** Average evaluation metrics for both models.

Model	MSE(+/-Std)	MAE (+/-Std)	RMSE(+/-Std)	Cos(+/-Std)	*P*-value	correlation	#rules(+/-Std)	time(+/-Std) in sec
ANFIS	0.0153(+/-0.007)	0.0813(+/-0.016)	0.12075(+/-0.031)	0.1293(+/-0.06)	0.00019	0.68279	32	256.50(20.9)
ANFIS-BPSO	0.0173(+/-0.015)	0.0746(+/-0.021)	0.12249(+/-0.053)	0.1328(+/-0.08)	0.00518	0.64768	29.2(+/-1.09)	80.963(+/-11.9)

**Fig. 3:**
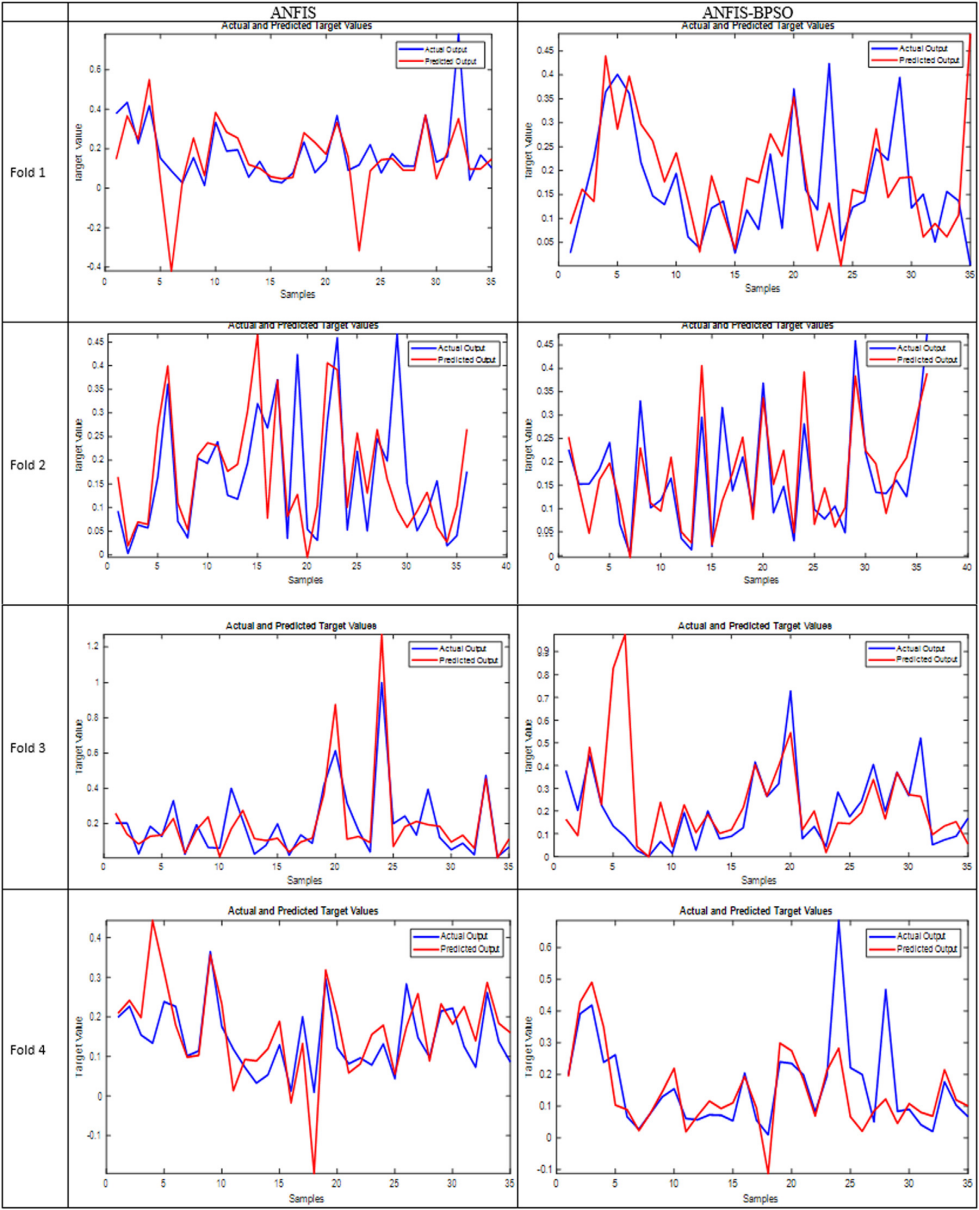

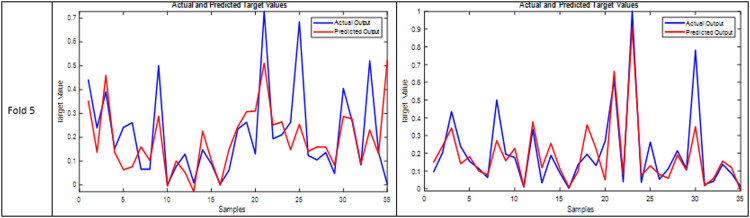
The performance Assessment plot of both models in each fold.

The ANFIS classifier has demonstrated a considerable impact on forecasting the Infarction Growth Rate (IGR). [7] has previously reported successful predictions of the IGR and infarction volume of the third CT scan utilizing ANFIS with no significant statistical differences from the ground truth (*P* = 0.489). To minimize the required CT scans, [8] proposed using ANFIS in conjunction with PCA to predict the second infarction growth rate from a reduced dataset. Our study focused on selecting the most significant features by calculating the *p*-value and the correlation coefficient between each feature and the IGRII, identifying up to five noteworthy features, as previously mentioned. Both ANFIS and ANFIS-BPSO models exhibited superior performance, as is clear in Fig. 3, which represents the evaluation assessment plot that shows the performance of both models in predicting the IGR II. In this figure, it is obvious that in each fold, both models show a similar pattern to the original target with a slight error in prediction for some samples. Despite this perfectness, it is observed that there is a significant difference at a certain point (like shown in fold 3 with ANFIS-BPSO model). The observed discrepancy between predicted and actual values in fold three of the cross-validation results could be attributed to various factors. Outliers or anomalies within these samples (despite we removed some outliers but not 100 %) might be driving the disparity, while unique characteristics of these samples could make them challenging for the model to predict accurately. The potential data imbalance could lead to inaccurate predictions for specific target values. The importance of features might also vary across folds, impacting predictions.

The improvement in this situation of ANFIS-BPSO over conventional ANFIS is represented by the training time and the number of generated rules, proving our aim of the experiment. The training time was reduced by nearly one-third of the original time, which is a crucial consideration for healthcare applications because faster training times for models enable quick and accurate diagnoses, timely interventions during emergencies, and efficient utilization of large and complex medical datasets. Shorter training times optimize workflows, improve patient experiences, and aid resource allocation. They also facilitate iterative improvement, adaptation to evolving medical data, and streamlined prototyping and testing, ultimately enhancing patient care, safety, and treatment personalization.

The generated rules may include both relevant and non-relevant ones. Reducing the number of generated rules may enhance performance, even if only a few are eliminated just like in our case for conventional ANFIS generated 32 rules, while ANFIS-BPSO reduced the number to approximately 29. The objective is to enhance the performance and processing time with significant rules, not only reduce the number of rules, and the ANFIS-BPSO model succeeded in this regard.

In terms of the other evaluation metrics, the results are very close for most of them. For some others, like MAE, we found that its amount for ANFIS-BPSO, which achieves around 0.07, is even better than Conventional ANFIS, which achieves around 0.08, as shown in Table 2. Furthermore, the *p*-value between the predicted value of both experiments with the original target was significantly lower (approximately 0.0001 for ANFIS and 0.005 for ANFIS-BPSO) compared to [7], who achieved only 0.489.

In the context of our use of ANFIS models, despite having a relatively modest number of features (initially 11, reduced to 5), we achieved reduction in dimensionality. While the term traditionally refers to challenges posed by high-dimensional feature spaces, we employed it here to emphasize the potential increase in model complexity arising from interactions and combinations of features, which can lead to computational challenges and overfitting.

### Comparison with similar technique

As discussed in the dataset section, it is not publicly accessible. This restriction prevents us from making a direct comparison with other works. Nevertheless, a comparison was made with the approach proposed by Ali et al. [8], for two primary reasons. Firstly, their research objective is aligned with ours, focusing on the prediction of IGR II. Secondly, they utilized a dataset akin to ours. Table 3 provides the comparative results considering Root Mean Square Error and Cosine distance, as these were the only evaluation metrics shared between our study and theirs.

**Table 3:** Comparison results between our models and other references.

Refs.	Description	RMSE	CosDistance
[8]	without PCA	0.439	0.616
[8]	with PCA	0.196	0.464
Proposed Model 1	ANFIS using highly correlated features	0.1266	0.1293
Proposed Model 2	ANFIS-BPSO using highly correlated features	0.1439	0.1328

Table 3 presents the comparative outcomes with [8], where both models demonstrated superior performance regarding RMSE and Cosine distance. There is a marginal distinction between the two, with the conventional ANFIS model achieving the lowest RMSE of 0.1266, compared to 0.143 for ANFIS_BPSO. This minor disparity reinforces the point that the BPSO-optimized ANFIS might exhibit either a marginal performance drop or increment compared to the traditional ANFIS, but this occurs within a significantly reduced training time, approximately halved, and a decreased rule generation volume. This claim is further substantiated by the Cosine distance values in Table 3.

### Comparison with other machine learning techniques

In addition to the forementioned points, a comparative study was conducted to substantiate the efficacy of our proposed model, ANFIS-BPSO, which we have previously demonstrated to outperform traditional ANFIS. The comparison was made with a set of distinct machine learning techniques that include Support Vector Regression (SVR), Shallow Neural Network (Shallow-NN), and Linear Regression (Lin-Reg). These models were tested on the same dataset as our proposed model; the only change is we compared ANFIS_BPSO when its membership function type is Gaussian. This change was based on several experiments on different types of membership functions to reach the best performance. The outcomes of this comparison, utilizing the evaluation metrics introduced in this study, are presented in Table 4.

**Table 4:** Average Evaluation metrics for comparing ANFIS and ANFIS-BPSO with other machine learning techniques.

Model	MSE	MAE	RMSE	CosDistance	*P*-value	correlation
ANFIS	0.0086	0.0655	0.0917	0.0764	7.9915e-06	0.8158
ANFIS_BPSO	0.0086	0.0643	0.0910	0.0743	3.8938e-06	0.7897
SVR	0.0087	0.0573	0.0907	0.0759	1.2704e-06	0.7850
Shallow-NN	0.0077	0.0595	0.0859	0.0740	9.8714e-06	0.7858
Lin-Reg	0.0083	0.0634	0.0903	0.0728	7.8396e-07	0.8093

**Fig. 4:**
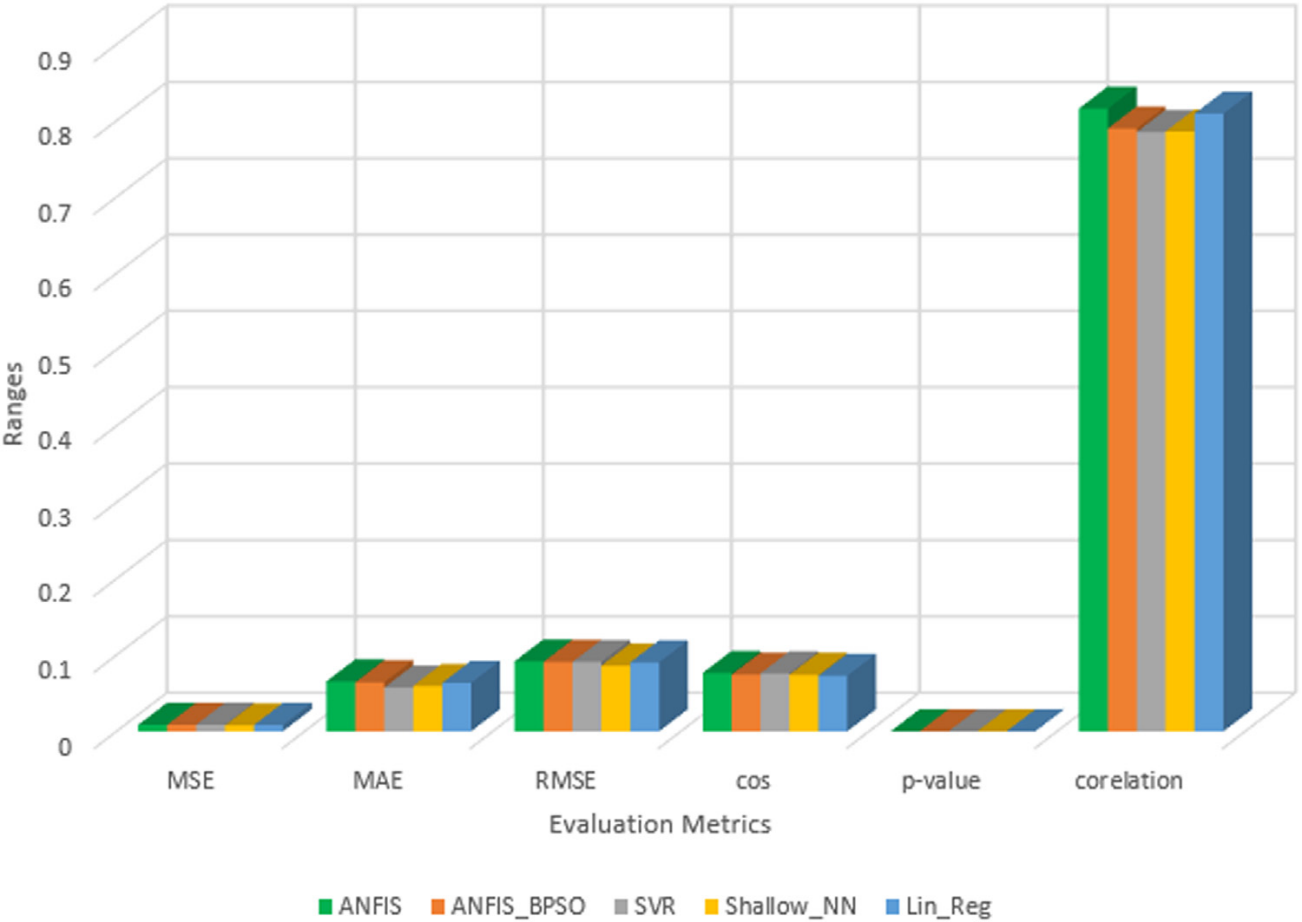
The comparison results among the ANFIS-BPSO and the rest of machine learning techniques.

Table 4 delineates the performance of both ANFIS and ANFIS-BPSO compared to other machine learning techniques, utilizing all the evaluation metrics. After several trials of several types of membership functions, we found that our proposed model as well as the conventional ANFIS perform optimally on our dataset using the Gaussian membership function to achieve results closely aligns with other machine learning models. Particularly, Shallow-NN yielded the lowest values for MSE, MAE, and RMSE at 0.0077, 0.0595, and 0.0859, respectively, while Lin-Reg exhibited the best Cosine distance and *P*-value at 0.0728 and 7.8396e − 07, respectively. Conventional ANFIS achieved the highest correlation coefficient at 0.8158. Fig. 4 offers a visual representation of the comparison between all models concerning all evaluation metrics, indicating a minor disparity between their performances.

Regarding the performance of our optimized model ANFIS-BPSO compared with Conventional ANFIS, it can be noticed that both models achieved the same MSE. Regarding all the rest of evaluation metrics, the ANFIS-BPSO outperformed ANFIS except with the correlation coefficient where the conventional ANFIS achieved the highest value compared with all other models in Table 4. We achieved these good performance of ANFIS-BPSO in 23 s compared with Conventional ANFIS which required 141.9 s and in less number of generated rules.

It's important to note that while the performance values across different models are similar, using ANFIS-BPSO provides benefits beyond just performance metrics. While other methods might provide results in less time, the choice of model should not be determined solely by speed but also by considering factors like interpretability, adaptability, and noise handling [[11], [24], [25], [26]]. Here are a few reasons why rule-based models like ANFIS can be a good choice:

• Interpretability: ANFIS models generate a set of understandable rules, which makes them highly interpretable. On the other hand, while Linear Regression is interpretable due to its straightforward relationship between inputs and outputs, SVR and Shallow Neural Networks, particularly, are often considered "black box" models. Interpreting their internal workings or the relationships they learn between inputs and outputs is challenging.
• Noise handling: ANFIS models, being rule-based and fuzzy, can handle noise in data better than Linear Regression, which can be sensitive to outliers. SVR has some capacity to handle outliers due to a margin, and Neural Networks can also handle noise to some extent. Still, they may require additional regularization techniques to avoid overfitting.
• Adaptability: ANFIS can learn and modify its rules during training. While SVR, Shallow Neural Networks, and Linear Regression models can adapt to the training data, they do not provide explicit rules that can be easily modified or interpreted.
• Knowledge Incorporation: ANFIS models can use domain knowledge as rules. This is not straightforward in SVR, Neural Networks, or Linear Regression.
• Non-linearity: While SVR and Neural Networks can handle non-linear relationships between inputs and outputs, Linear Regression can only model linear relationships unless extended with additional features. ANFIS, based on fuzzy logic, can inherently model non-linear relationships.

In summary, ANFIS offers a combination of interpretability, adaptability, and effective handling of noise and non-linearity, which makes it advantageous in scenarios where these qualities are desirable. The only limitation is that its training time is still high compared with the other machine learning techniques utilized in this study.

## Funding statement

This work is not funded by any agency.

## CRediT authorship contribution statement

**Afnan Al-Ali:** Conceptualization, Methodology, Software, Validation, Formal analysis, Writing – original draft, Writing – review & editing, Visualization. **Uvais Qidwai:** Supervision, Investigation, Resources, Writing – review & editing. **Saadat Kamran:** Resources, Data curation.

## Declaration of Competing Interest

The authors declare that they have no known competing financial interests or personal relationships that could have appeared to influence the work reported in this paper.

## Data Availability

The authors do not have permission to share data.
